# The proteome profiles of the olfactory bulb of juvenile, adult and aged rats - an ontogenetic study

**DOI:** 10.1186/s12953-014-0058-x

**Published:** 2015-02-15

**Authors:** Michael Wille, Antje Schümann, Michael Kreutzer, Michael O Glocker, Andreas Wree, Grit Mutzbauer, Oliver Schmitt

**Affiliations:** Department of Anatomy, Gertrudenstr. 9, 18055 Rostock, Germany; Proteome Center Rostock, Schillingallee 69, 18055 Rostock, Germany; Department of Pathology, Josef-Schneider-Str. 2, 97080 Würzburg, Germany

**Keywords:** Proteomics, Rat, Brain, Olfactory bulb, Development

## Abstract

**Background:**

In this study, we searched for proteins that change their expression in the olfactory bulb (oB) of rats during ontogenesis. Up to now, protein expression differences in the developing animal are not fully understood. Our investigation focused on the question whether specific proteins exist which are only expressed during different development stages. This might lead to a better characterization of the microenvironment and to a better determination of factors and candidates that influence the differentiation of neuronal progenitor cells.

**Results:**

After analyzing the samples by two-dimensional polyacrylamide gel electrophoresis (2DE) and matrix-assisted laser desorption/ionization time-of-flight mass spectrometry (MALDI-TOF-MS), it could be shown that the number of expressed proteins differs depending on the developmental stages. Especially members of the functional classes, like proteins of biosynthesis, regulatory proteins and structural proteins, show the highest differential expression in the stages of development analyzed.

**Conclusion:**

In this study, quantitative changes in the expression of proteins in the oB at different developmental stages (postnatal days (P) 7, 90 and 637) could be observed. Furthermore, the expression of many proteins was found at specific developmental stages. It was possible to identify these proteins which are involved in processes like support of cell migration and differentiation.

**Electronic supplementary material:**

The online version of this article (doi:10.1186/s12953-014-0058-x) contains supplementary material, which is available to authorized users.

## Background

The aim of this study was to analyze the differential proteome of the rat olfactory bulb (oB) within different developmental stages (7-day-old juvenile rats (P7), 90-day-old adult rats (P90) and 637-day-old aged rats (P637)).

The postnatal development of the oB corresponds to a bulging of the cerebral hemispheres. It develops from the cerebral vesicle near the placode. At the time of birth, the oB is one of the most rostral regions of the rat brain [1].

In addition, the oB integrates different neuronal microcircuits which are of fundamental physiological importance for olfaction. It has a particular source of sensory input (axons from olfactory receptor neurons of the olfactory epithelium) and one output source (mitral cell axons) which work as a filter to enhance the sensitivity of odor detection and its information processing [2]. The sensory input is created by axons from olfactory receptor neurons of the olfactory epithelium; the output is organized by the mitral cell axons. Additionally, information can also be received from other parts of the brain (amygdala, neocortex, hippocampus, locus coeruleus and substantia nigra). The peripheral olfactory system has also been recognized as a model of postnatal neurogenesis, because it displays the most robust and functional postnatal neurogenesis among neuronal populations that maintain stem cells [3]. In addition, neurogenesis is increased after lesioning of either the oB (termed bulbectomy) or the epithelium itself [4]. The epithelium contains a mixed population of basal cells, basal daughter cells, immature and mature olfactory receptor neurons (ORNs), and sustentacular cells [5,6]. Basal cells and their daughter cells generate the new neurons that repopulate the olfactory epithelium (OE) throughout life [7-10]. The presence of stem cells in the adult brain which can develop into mature neurons in vitro suggests that postnatal neurogenesis could be used to repopulate certain neuronal lineages [11,12]. Therefore, different factors for neuronal precursor proliferation and maturation have been determined (including neurotrophins, neuropeptides, and cytokines [13,14]). Despite the usage of certain mitogens, the proliferation of neuronal stem cells can be induced in vitro. The identification of factors that regulate and modulate the maturation of neurons within the postnatal brain is critical to the application of stem cell therapy [15-17].

According to Bandeira et al. [1], a postnatal increase of the rat brain-mass occurs in the first 3 months after birth (by a factor of ~20.7 in all brain areas). The time course of changes in the postnatal brain can be divided into 4 phases, the first 2 phases (first phase [P0-P2]: “idle time“, no significant mass increase of the brain; second phase [P2-P25]: heavy increase of the brain’s mass) can be referred to all brain structures. Furthermore, from P25 on, the hippocampus, oB and the rest of the brain show a slow growth up to P90. The weight of body and brain increases till P275, but much slower with rising age [18].

**Figure 1:**
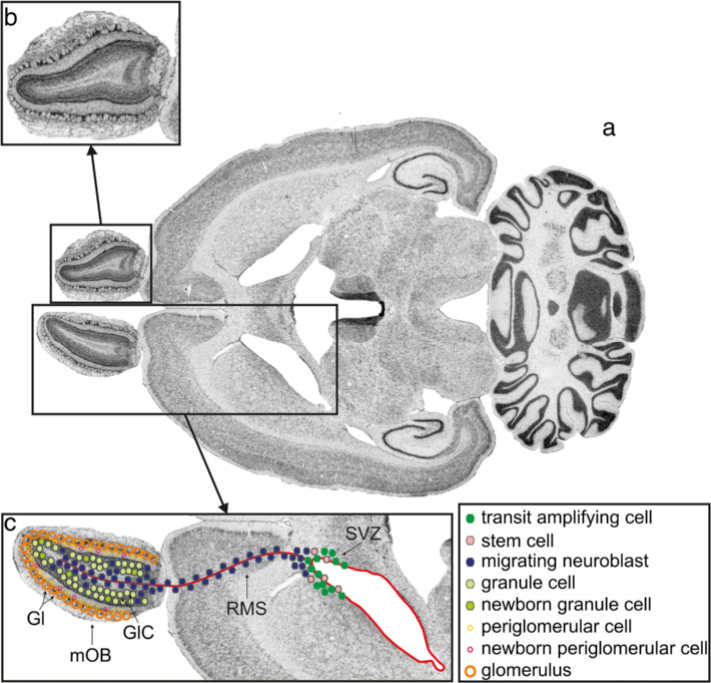
**Overview of the rat brain at the level of the olfactory bulb. (a)** Horizontal section of a rat brain, **(b)** Magnified view of the olfactory bulb. **(c)** Schematic overview of the migration of olfactory precursor cells (RMS).

For the postnatal changes in the number of neurons, the rat brain generates between 1 and over 5 million neurons per day during a period of 3–4 days. During the first postnatal week, then, the number of neurons increases by nearly 50% in the brain. At this stage, the number of neurons is followed by a marked net loss of neurons during the second postnatal week, when 60%–70% of the number of neurons in the brain is lost. In contrast, an addition of neurons proceeds in the oB until adulthood. An identifiable period of neuronal loss within adulthood is not detectable in this brain area. The generation and integration of new neurons is possible because the rostral migratory stream (RMS) of the subventricular zone (SVZ) at the anterior horn of the lateral ventricles progressively fills up and replaces neurons of the oB which degenerate [19-23]. It presents an active neurogenerative region in adulthood (schematic overview, see Figure 1c). Here, the progenitor cells proliferate continuously and differentiate to granular and periglomerular cells in the oB [23-27]. These progenitor cells migrate from the SVZ to their functional destination in the oB. Studies by Luskin [27], Lois and Alvarez-Buylla [23] and Luskin and Boone [28] identified the migratory route as a pathway where the velocity of migration constitutes 5 mm/7 days in the adult rat and mouse between the anterior horn of the lateral vesicle and the olfactory ventricle. The cells spread radially towards the granular and periglomerular layers, where they are thought to differentiate into neurons [23,27,29]. In adult rats, this subependymal layer is a remnant of the embryonic subventricular zone which originates after the occlusion of the primitive olfactory ventricle where cell proliferation can occur in the adulthood. This proliferation is organized in a well-defined pathway of tangential and radial migration. The migration is performed by a meshwork of astrocytic processes and cell bodies which form long tangentially oriented canals. These canals act as physical barriers, allowing the deplacement of long chains of migrating cells from the lateral ventricle to the oB and prevent any dispersion along their tangential migration route. Interestingly, cells inside these glial tubes contain proteins like class 3 beta-tubulin [30]. This beta-tubulin shows a diffuse staining which signals a not yet organized form of mature finally assembled microtubules [26]. With this mechanism, it is possible for neurons as well as for progenitor cells (differentiated in the stem cell niche) to migrate within the oB. The progenitor cells can differentiate to dopamineric interneurons. The main period of origin of interneurons is located between P1 and P10, after this period their development decreases. This means the chosen period of P7 in this study can be seen as one of the main peaks of generating interneurons.

Another major amount of olfactory interneurons are the granular cells. This GABAergic cell type is important for generating inhibition to regulate the processing of sensory output. These cells are essential for learning and for the manipulation of sensory experiences early in the postnatal period. They are able to modify the structure and function of the oB [31-34]. Regarding their morphology, these cells are axonless interneurons and their dendrites span several bulbar layers and have distinct layer-specific features [35]. Furthermore, granular cells run through considerable changes of their formation of spines and filopodias. During development, three major changes are known: decrease of stubby spines, accompanied by an increase in the frequency of typical spines, then a distinct pattern of change in spine, filopodium density in the different dendritic domains and an overshoot in the number of spinesor filopodia, respectively, in the basal dendrites (peak at P28).

A main aspect during growth and development of the rat brain are the expression- and activity changes of glycolytic enzymes. For example, the activity of phospho-fructokinase (a key enzyme which catalyzes the conversion of fructose-6-phosphate to fructose-1, 6-bisphosphate and which also represents the rate-limiting step in the glycolysis) is detectable in fetal brains at the twelfth day of gestation. It shows little change in enzyme activity in the brain from 5 days before birth to 8 day after birth. During this period, activity was 30-40% of the normal value found in an adult brain. After 12 days, a rapid increase in activity to 21 days occurred [36]. Furthermore, the activities of additional glycolytic enzymes (hexokinase [37], aldolase [38], lactate-dehydrogenase [39]) do show substantial increases during maturation of the rat brain.

A main feature of Parkinson’s disease is the loss of dopaminergic neurons in the substantia nigra [40]. Current therapeutic options of this neurodegenerative disease are symptomatic and temporary only. The experimental investigation of optimizing therapeutic strategies like stem cell transplantation in well described experimental models of Parkinson’s disease, both in vitro and in vivo are still essential [41]. From previous studies [42,43], we know that transplanted progenitor cells in the neonatal and adult striata develop differently in neural and glial cell types. A better characterization of the development of the microenvironment at the proteome level may support a better understanding of survival and differentiation of transplanted progenitor cells.

In this study, proteins from the oB of juvenile, adult and aged rats were separated by 2DE and identified by MALDI-TOF-MS. Proteins which change their expression during ontogenesis were identified. As yet, protein expressions in the developing proteome are not fully understood. As mentioned before, stem cells of the oB in the adult brain can develop into mature neurons in vitro which suggests that postnatal neurogenesis could be used to repopulate certain neuronal lineages [11,12]. Additionally, with the usage of certain mitogens and cytokines, the proliferation of neuronal stem cells can be induced [15-17]. Based on this, the oB seems an interesting system to analyze differences in the expression of proteins depending of its development.

## Results

To identify the differences in the protein expression between the groups of different ages (P7, P90 and P637), 2DE with subsequent gel-matching, spot-warping and differential spot analysis combined with protein identification by MALDI-TOF-MS was performed. A precise, manual labeling of the spots was realized by using the software Progenesis PG200.

First, in Figure 1a, a horizontal section of the oB of a rat brain is shown. In Figure 1b, a magnified view of the analyzed brain area (oB) is given. In Figure 1c, a schematic overview of the migration of olfactory precursor cells is presented. Here, the olfactory precursor cells (stem cells, transit amplifying cells) proliferate mainly in the SVZ where the differentiation in immature neuroblasts also happens. These neuroblasts then migrate through the RMS to the oB. After a period of 5–7 days (after birth), these neuroblasts shift towards the granular (granular cell layer, GlC), periglomerular and external plexiform cell layers of the oB. At a time of 15–30 days after birth, matured neuroblasts generate interneurons in the oB.

**Figure 2:**
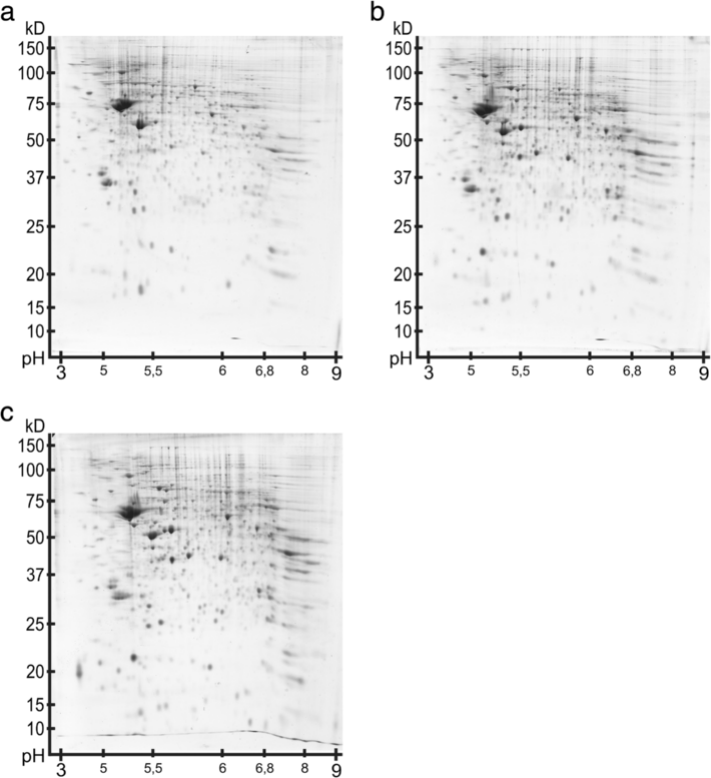
**Overview of the reference gel images from the olfactory bulb.** The coomassie blue stainings of the gels have similiar intensities. The 2DE-gel image of a P7 animal is shown in **(a)**. In **(b)** the 2DE-gel image of a P90 animal is presented. **(c)** shows the 2DE-gel image of an oldest P637 rat.

**Figure 3:**
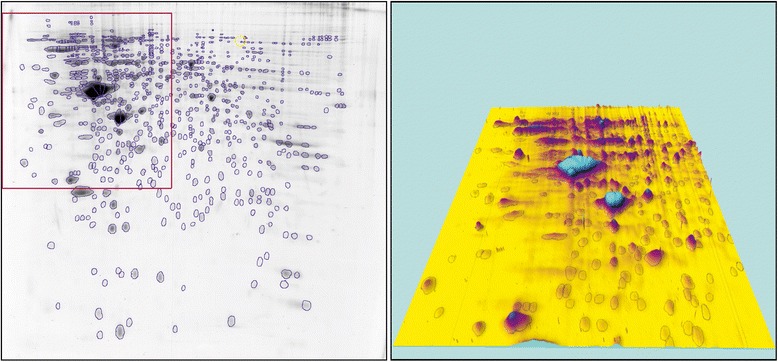
**In order to perform an accurate segmentation of spots, the manual editing of spots was augmented by a 3D-visualization.** This procedure has been applied to all reference gels as well as to all template gels. An example of the latter is presented here.

In Figure 2, the images of the reference gels of the different developmental stages are presented ((a) P7, (b) P90 and (c) P637). The spot compositions turn out to be comparable. On average, 828 (±71) spots were detected in the six gels of P7, 775 (±152) spots in the gels of P90 and 785 (±56) spots in the gels of P637. Furthermore, in Figure 3, an example of the manual spot editing (segmentation, delineation) by using a gel image of the olfactory bulb in Progenesis PG200 is shown.

**Figure 4:**
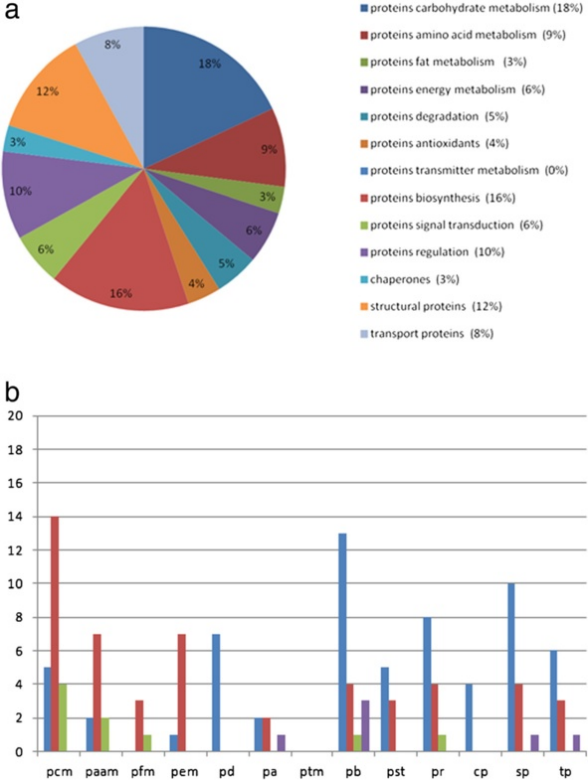
**Differential expression of proteins in the olfactory bulb at P7. (a)** Relative frequencies of proteins in the olfactory bulb that are differentially expressed (P7 vs P90). **(b)** Number of differentially expressed proteins of different protein categories within the olfactory bulb [P7] vs [P90]. (Abbreviations for Figure 4 (b) and Figure 5 (b): pcm: proteins carbohydrate metabolism, paam: proteins amino acid metabolism, pfm: proteins fat metabolism, pem: proteins energy metabolism, pd: proteins degradation, pa: proteins antioxidants, ptm: proteins transmitter metabolism, pb: proteins biosynthesis, pst: proteins signal transduction, pr: proteins regulation, cp: chaperones, sp: structural proteins, tp: transport proteins). Number of up-regulated proteins for each category are shown in blue; down-regulated proteins (red); absent proteins in P7 (green) and in P90 (purple).

**Figure 5:**
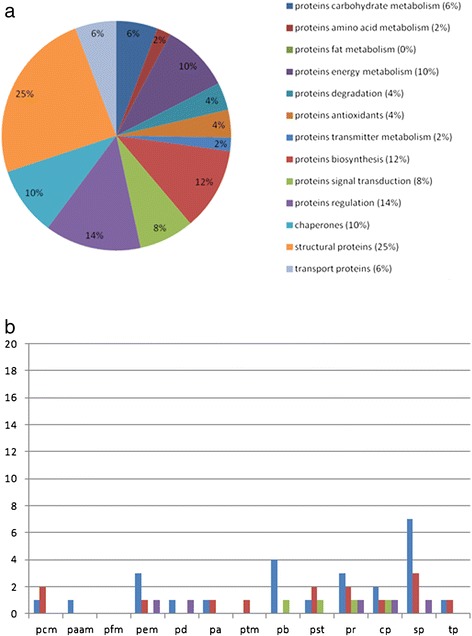
**Differential expression of proteins in the olfactory bulb at P637. (a)** Relative frequencies of proteins in the olfactory bulb that are differentially expressed (P637 vs P90). **(b)** Number of differentially expressed proteins of different protein categories within the olfactory bulb [P637] vs [P90]. Same abbreviations and labeling performed as in Figure 4 (b); absent proteins in P637 (green) and in P90 (purple).

The differentially expressed proteins were classified into 13 functional protein groups. In the following, only those proteins which show the most differential expression are described in detail (Figures 4(a) and 5(a)). A complete overview of the expression changes of all analyzed proteins as well as a description of the remaining protein categories is listed in Additional file 1.

In the following, a more detailed analysis of the up- and down-regulation of the proteins from the different categories and developmental stages in comparison to P90 is described (4(b), 5(b)).

**Figure 6:**
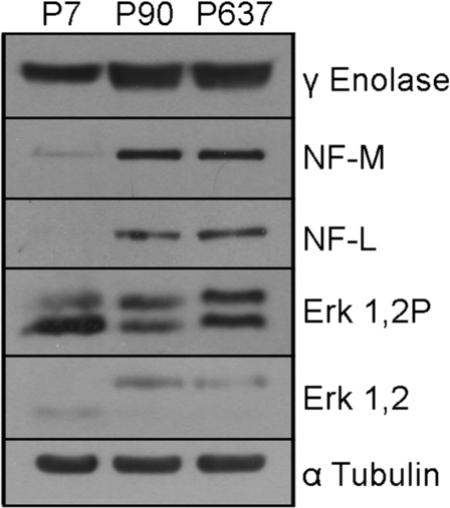
**The validation of some differially expressed proteins was performed by Western Blots (Mitogen-activated protein kinase 1, 2 (Erk1,2), Phospho Mitogen-activated protein kinase 1, 2 (Erk1,2P), Neurofilament Low (NF-L), Neurofilament Medium (NF-M) and Gamma Enolase (γ Enolase) in the olfactory bulb between the different development stages (P7, P90, P637). **α-tubulin staining was used to ensure the equal loading of proteins. This protein is expressed also differentially.

For P7, the carbohydrate metabolism consists of 14 proteins which are down-regulated in this stage towards P90. For example, the protein 2-oxoglutarate dehydrogenase (Ogdh) which catalyzes the overall conversion of alpha-ketoglutarate to succinyl-CoA and CO_2_ during the Krebs-cycle and which also participates in mitochondrial degradation of glutamate shows a down-regulation. Aconitate hydratase (Aco2) that catalyzes the interconversion of citrate to isocitrate via cis-aconitate in the second step of the TCA-cycle is down-regulated as well. Another down-regulated protein towards P90 is the gamma-enolase (Eno2) which has neurotrophic and neuroprotective properties on a broad spectrum of neurons. This expression pattern could also be validated by Western Blot analysis of the same protein homogenates (Figure 6). It binds in a calcium-dependent manner to cultured neocortical neurons and promotes the survival of the cells. Furthermore, proteins like glucose-6-phosphate 1-dehydrogenase (G6pdx), which produces pentose sugars for nucleic acid synthesis and which is a secondary antioxidant enzyme that plays an important role in detoxifying ROS/RNS by maintaining a supply of intermediates such as glutathione and NADPH are differentially expressed. Glyceraldehyde-3-phosphate dehydrogenase (Gapdh), a protein involved in the important energy-yielding step in carbohydrate metabolism, catalyzes the reversible oxidative phosphorylation of glyceraldehyde-3-phosphate and is also participating in the organization and assembly of the cytoskeleton. Altogether 5 proteins including alpha glucosidase (Ganab) with a hydrolytic function as well as transaldolase (Taldo1) are up-regulated at this stage. Taldo1 presents an important regulator of metabolites in the pentose-phosphate pathway, for example. Furthermore, 4 proteins are absent at this stage. For example, the protein 3-hydroxyisobutyrate dehydrogenase (Hibadh) plays a critical role in the catabolism of L-valine by catalyzing the oxidation of 3-hydroxyisobutyrate to methylmalonate semialdehyde. Fructose-bisphosphate aldolase C (Aldoc) which gets expressed specifically in the hippocampus and in Purkinje cells of the brain, catalyzes the reversible aldol cleavage of fructose-1.6-biphosphate and fructose 1-phosphate to dihydroxyacetone phosphate and either glyceraldehyde-3-phosphate or glyceraldehydes. Glycerol-3-phosphate dehydrogenase 1-like protein (Gpd1l) catalyzes the conversion of glycerol 3-phosphate to glycerone phosphate. The protein isocitrate dehydrogenase subunit alpha (Idh3a) catalyzes the oxidative decarboxylation of isocitrate to 2-oxoglutarate.

As for the proteins of the biosynthesis, 13 proteins are up-regulated. For example, the protein serine tRNA ligase (Sars) catalyzes the attachment of serine to tRNA. Additionally, different subunits needed for splicing (splicing factor 3a, subunit 3 (Sf3a3)), elongation (elongation factor 1-gamma (Eef1g)) and translation (eukaryotic translation initiation factor 4A1 (Eif4a1)) display an up-regulation. Furthermore, different members of the family of the heterogeneous nuclear ribonucleoproteins (hnRNPs) are up-regulated towards P90 (heterogeneous nuclear ribonucleoprotein (Hnrnpf), heterogeneous nuclear ribonucleoprotein C (Hnrnpc), heterogeneous nuclear ribonucleoprotein K (Hnrnpk)). These members are essential for mRNA metabolism, DNA-related functions and microRNA biogenesis. Four proteins are down-regulated towards P90. These proteins are involved in the metabolism of biosynthesis like the elongation factor Tu (Tufm) or serine/threonine-protein phosphatase 2A 55 kDa regulatory subunit B alpha (Ppp2r2a). One protein is absent in the gels from the P7 protein extract (ribose-phosphate pyrophosphokinase 1 (Prps1)) which catalyzes the phosphoribosylation of ribose 5-phosphate to 5-phosphoribosyl-1-pyrophosphate, necessary for purine metabolism and nucleotide biosynthesis. Three proteins are absent in the gels from the P90 protein extract: The serine/arginine-rich splicing factor 3 (Sfrs3), the RNA- binding motif protein, X-linked-like-1 (Rbmxl1) and the putative RNA-binding protein 3 (Rbm3), a protein which is expressed widely during early brain development in glutamatergic and GABAergic cells as well as in newly formed and migrating neurons, peaking in the first to second postnatal weeks.

The amount of up- and down-regulation of the structural proteins is more or less balanced. Thirteen proteins are up-regulated at this stage. These proteins are mainly involved in the dynamic organization of the cytoskeleton, like actin-related protein 2 (Actr2), member of the Arp2/3 complex, dihydropyrimidinase-related protein 2 (Dpysl2) or F-actin-capping protein subunit alpha-1 (Capza1). Other up-regulated proteins are actin beta (Actb) which can support the acceleration of axonal outgrowth, for example, or stathmin (Stmn1) involved in the regulation of the microtubule filament system by destabilizing microtubules. Six proteins are down-regulated towards P90. These are mainly members of the neurofilament family (neurofilament light polypeptide (Nefl), neurofilament medium polypeptide (Nefm)) which comprise the axoskeleton and functionally maintain the neuronal caliber. They may also participate in intracellular transport to axons and dendrites. This pattern of expression could also be validated by Western blot analysis for the neurofilament medium polypeptide as well as for the neurofilament light polypeptide (Figure 6). The tropomyosin alpha-3 chain is the only protein which is absent at P90 in this category. This protein has the ability to regulate the molecular composition of microfilaments, which in turn regulates the dynamic and functional properties of the resulting actin filament population.

All in all, at developmental stage P7, a total of 129 proteins is expressed differentially towards P90, of which 63 proteins are up-regulated, 51 proteins are down-regulated, 9 proteins are absent at P7 and 6 proteins are absent at P90 (Table 1).

**Table 1:** **Overview of the differential expression of the proteins in P7 and P637 vs. P90**

**Categories**	**Amount [P7] (absolute numbers)**	**Amount [P637] (absolute numbers)**
Total	129	47
Upregulated	63	25
Downregulated	51	14
Absent	9	4
Absent P90	6	4

For P637 in comparison to P90, differences in the expression are also remarkable. Beginning with the functional group with the most differentially expressed proteins, the structural proteins; this category consists of 7 proteins which are up-regulated, 4 proteins which are down-regulated. The up-regulated protein faction contains actin beta (Actb) and myristoylated alanine-rich C-kinase substrate (Marcks) which also shows an up-regulated expression at P7 towards P90. Other up-regulated proteins are members of the tubulin family (tubulin alpha-1A chain (Tuba1a), tubulin beta-3 chain (Tubb3), tubulin beta 4A (Tubb4)). These proteins are generally required for the organization of the cytoskeleton as well as migration and differentiation of neurons, for example, by assembling microtubules into a highly organized mitotic spindle. The down-regulated proteins contain lamin-B1 (Lmnb1) and septin-11 (Sept11), for example. The protein Lmnb1 (involved in nuclear stability, chromatin structure and gene expression) shows an up-regulation at P7 towards P90 and Sept11 (filament-forming cytoskeletal GTPase, involved in cytokinesis and vesicle trafficking) displays a down-regulation at P7.

As for the regulatory proteins, 3 proteins are up-regulated. These contain the Rab GDP dissociation inhibitor alpha (Gdi1) which is also up-regulated at P7 in comparison to P90. Another protein is the Rap2-interacting protein × (Rufy3) which is implicated in the formation of a single axon of developing neurons. A total of 2 proteins (Rho GDP-dissociation inhibitor 1 (Arhgdia), annexin A5 (Anxa5)) is down-regulated. Arhgdia is involved in the organization of the actin cytoskeleton in response to extracellular signals for proper cell morphology, growth, proliferation, differentiation, motility and adhesion. The protein Anxa5, whose functional meaning was described earlier, is also down-regulated at P7 towards P90. One protein is absent at P637 (calcium binding protein 39 (Cab39)). This protein presents a necessary element in cell metabolism that is required for maintaining energy homeostasis. At P90 the protein myosin regulatory light chain 12B (Myl12b) is absent.

The proteins which are involved in pathways of the protein biosynthesis present 4 proteins which are up-regulated and 1 protein which is absent at P637 (transcriptional activator protein Pur-beta (Purb)). The up-regulated group contains members of the heterogeneous nuclear ribo-nucleoprotein family (heterogeneous nuclear ribonucleoprotein F (Hnrnpf), heterogeneous nuclear ribonucleoprotein K (Hnrnpk)). These proteins are relevant for mRNA metabolism, DNA-related functions and microRNA biogenesis.

At P637, all in all 47 proteins are expressed differentially towards P90, of which 25 proteins show an up-regulation, 14 proteins show a down-regulation, 4 proteins are absent at P7 and 4 proteins are absent at P90 (Table 1).

## Discussion

In the following, the identification and characterization of differentially expressed proteins in the oB during the postnatal day of development (P7 and P637) in comparison to P90 will be discussed.

In general, the oB does not consist of a homogeneous cell population. Therefore, a differential proteome analysis is a representation of proteome changes of the whole oB concerning all cell populations and the neuropile. The separation technique used in this study allows protein separation within the range of approximately 10–100 kDa and between a pH-range of 3–10. For this reason, it is possible to analyze a substantial part, but not the entire proteome of the oB [44]. Moreover, some parts of the gel may show a lower resolution which can lead to a misinterpretation of the regulation analysis of some protein spots. Furthermore, it is known that proteins may be represented by multiple spots with different locations in the gel which can result from different posttranslational modifications and isoforms of the same protein. Therefore, several proteins may show specific regulation changes for one spot only and not for the entire protein. By applying other mass spectrometric detection methods like the stable isotope ratio mass spectrometry (SIRMS), it would be possible to unambiguously identify both the protein and its variants and to overcome the sample-to-sample recovery variabilities associated with non-SIRMS MS-proteomic methods. Moreover, the usage of affinity-enrichment-MS methods (e.g. IDBEST™, iTRAQ™) would enhance the possibility for targeted biomarker discovery applications to drill down to lower-abundance proteins and to improve its analysis [45]. Hence, the results of the presented proteome analysis of the rat oB should be considered as a first step to elucidate the patterns of proteins which are differentially expressed in this region of the brain.

This study focuses on proteins that differed in abundance and give indications for developmental changes in the oB. Expression levels of proteins were determined and their changes were interpreted by comparison with regard to the literature. Specific changes of cytoplasmatic proteins of the postnatal developing oB were found.

At P7 in comparison to P90, those proteins which are involved in carbohydrate metabolism, biosynthesis, amino acid metabolism and regulatory as well as structural proteins show an increased expression. For the protein expression at P637 versus P90, proteins of the energy metabolism, proteins involved in biosynthesis, chaperones, regulatory and structural proteins show various changes in their expression.

The majority of differentially expressed proteins of the carbohydrate metabolism are down-regulated at P7 with comparison to P90. Most of these proteins are involved in the tricarboxylic acid cycle and glycolysis. For example, the protein 2-oxoglutarate dehydrogenase (Ogdh) which catalyzes the conversion of alpha-ketoglutarate to succinyl-CoA and CO_2_ during the Krebs-cycle and which also participates in mitochondrial degradation of glutamate [46] shows a down-regulation at P7. Other proteins like aconitate hydratase (Aco2) participating in the interconversion of citrate to isocitrate or the protein gamma-enolase (Eno2) which catalyzes the conversion of 2-phosphoglycerate to phosphorenolpyruvate and which also has neuroprotective properties [47] are involved in these pathways. Already Wilbur et al. [48] found that the activities of several proteins involved in tricarboxylic acid cycle as well as of the pyruvate metabolism show low expression levels in newborn rats, whereas they are increased markedly to adult levels during 10–30 postnatal days but do not decrease in adulthood.

The majority of proteins which are involved in the processes of protein biosynthesis display an up-regulation at P7 in comparison to P90. Especially proteins for splicing (splicing factor 3a, subunit 3 (Sf3a3)), elongation (elongation factor 1-gamma (Eef1g)) and translation (eukaryotic translation initiation factor 4A1 (Eif4a1)) are up-regulated. Of particular relevance in the mammalian nervous system are transcripts exhibiting multiple splicing patterns, particularly important growth processes such as axon guidance, synaptogenesis, and the regulation of membrane physiology [49-51]. Furthermore, different members of the family of the heterogeneous nuclear ribonucleoproteins (hnRNPs) are up-regulated towards P90 (heterogeneous nuclear ribonucleoprotein (Hnrnpf), heterogeneous nuclear ribonucleoprotein C (Hnrnpc), heterogeneous nuclear ribonucleoprotein K (Hnrnpk)). These members generally play key roles in mRNA metabolism, DNA-related functions and microRNA biogenesis. In general, regulation of stability, localization and translation of a number of transcripts are important in the establishment and maintenance of nerve cell functions [52-56]. The regulation of the expression of many RNAs depends on the activity of RNA-binding proteins, often acting in concert and organized in complexes, probably regulating the activity and the expression of the other components of the complex itself [57]. These complexes contain elements involved in the control of different aspects of RNA metabolism, including the interactions established by heterogeneous nuclear proteins (hnRNPs) during the transport of the mRNAs from nucleus to cytoplasm and to the site of translation. They also contain mRNA–proteins and pre-mRNP complexes which can be actually found in association with the cytoskeleton. Models have been proposed about the role of cytoskeleton-associated hnRNPs in mRNA biogenesis-control at the post-transcriptional level [58,59]. For example, hnRNPK and the Abelson-interacting protein 1 (Abi-1) can act in a synergistic manner in a multiprotein complex that regulates the crucial balance between filopodia formation and synaptic maturation in neurons [60].

Especially proteins for the dynamic organization of the cytoskeleton are up-regulated at the early developmental stage of P7, as, for example, the actin-related protein 2 (Actr2) which is a member of the Arp2/3 complex. This complex is a stable assembly of two actin-related proteins (Arp2 and Arp3) and subsequently five other proteins. This nucleator acts by serving as a template for monomer addition by mimicking the barbed end of a growing filament [61]. This complex gives rise to branched actin filaments, as the complex binds to the sides of actin mother filaments and remains associated with the pointed end leaving the barbed end free to elongate [62,63]. Other up-regulated proteins (F-actin-capping protein subunit alpha-1 (Capza1), actin beta (Actb), stathmin (Stmn1)) are also involved in the dynamic organization which can support neuronal differentiation, including axonal growth and branching, or dendritic development [64]. In addition, Kaltwasser et al. [65] and Stühler et al. [66] reported that with increasing age a down-regulation of Stmn1 could be observed in the rat brain. Some members of the neurofilament family are down-regulated (neurofilament light polypeptide (Nefl), neurofilament medium polypeptide (Nefm)). These are involved in the promotion of axonal growth, neuronal polarity through direct binding to tubulin and known to be required for signaling in axon guidance [67]. As McAllister [68] described, dendritic growth and its arborization are crucial for the proper postnatal development of the nervous system. It suggests that levels of activity at individual synapses can locally modify the structure of the postsynaptic neuron. Furthermore, most of the extra- and intracellular signals that influence dendritic growth also alter synapse formation and axon guidance. This means that especially in the first postnatal days, the development of neurons and formation of synapses e.g. is essential, therefore, a dynamic expression of structural proteins is important at this stage of development.

A comparable development expression is also detectable for regulatory proteins. This category includes 8 up-regulated proteins and 4 down-regulated proteins. Cofilin-1 (Cfl1) and neuromodulin (GAP-43) among others belong to the up-regulated proteins at P7. Cfl1 has the property to directly regulate actin dynamics, severing and depolymerizing actin filaments to generate new barbed ends for an initiation of the actin polymerization. One of the best characterized ways of regulation is the phosphorylation on serine 3 that inhibits its F-actin activity [69]. Moreover, Sparrow et al. [70] showed, that cofilin plays an essential role during myelination in combination with neuregulin-1. This presents a highly specialized form of cell motility in which protrusive expansion of the leading edge of the inner mesaxon, accompanied by high rates of membrane synthesis, drives the glial membrane repeatedly around the axon to generate the myelin sheath. As stated by Philpot et al. [71], a proper formation of myelin has important consequences for the physiological function. They were able to show that an increased myelination occurs in the oB beginning about P11 and continues through P30. These results are corroborated by the appearance and rapid increase in expression of myelin-specific mRNAs in the rat bulb from P10-P15 [72]. The protein neuromodulin (GAP-43) presents a neuron-specific phosphoprotein that appears to be participating in the development and functional modulation of synaptic relationships [73]. During development, GAP-43 is one of a small number of proteins that is expressed selectively in association with process outgrowth [74]; its synthesis and axonal transport persist at high levels throughout axogenesis and synaptogenesis, and then decline precipitously with the establishment of stable synaptic relationships [75-81]. Jacobson et al. [82] observed that throughout the rat brain as a whole, levels of GAP-43 are highest during the first postnatal week, a period in which synaptic organization is still taking place, then fall by more than 90% over the next few weeks. For the down-regulated proteins, e.g., members of the annexin family (annexin A3 (Anxa3), annexin A5 (Anxa5)) show a lower amount of expression compared to P90. An accumulation of annexin 5 in cultured glioma cells during differentiation rather than proliferation could be shown by Giambanco et al. [83]. It is known that brain development and maturation in the rat are postnatal events, consisting of glial cell proliferation and differentiation after the tenth postnatal day and in synaptogenesis during the third and fourth postnatal week [84-86]. Furthermore, Giambanco et al. [83] provided evidence that the accumulation of annexin 5 shows a sharp increase after the first postnatal week which appears to be in line with the possibility that the expression of this protein might be related to brain development.

The proteins of the amino acid metabolism show a higher amount of down-regulated proteins (7 proteins) than up-regulated proteins (2 proteins). The protein GMP synthase (GMPS) is an example for an up-regulated protein in comparison to P90 in this category. GMP synthetase is a key enzyme in the de novo synthesis of guanine nucleotides [87]. In the guanine nucleotide pathway, two enzymes are involved in converting IMP (inosinmonophosphat) to GMP. Here, GMP synthetase catalyzes the amination of XMP (Xanthosinmonophosphat) to GMP. The accumulation of guanine nucleotides is not only essential for DNA and RNA synthesis, but it also provides GTP, which is involved in a number of cellular processes important for cell division. GTP hydrolysis is also required for protein glycosylation [88], synthesis of adenine nucleotides [89], protein translation [90], activation of G-proteins [91] and microtubule assembly [92] which presents a possible explanation for the up-regulated expression of the protein at P7 compared to P90. A dynamic organization of the cytoskeleton is essential for neuronal differentiation (axonal growth and branching, dendritic development) in the early stages of development. Furthermore, a higher amount of down-regulated proteins was detectable at this postnatal developmental stage for the regulatory proteins. As mentioned earlier, the protein glutamine synthetase (Glul) displays a lower expression at P7 than at P90. Also Patel et al. [93] presented a differential pattern within development for this protein in different brain regions. At birth, the protein activity was the highest in the oB followed by forebrain and cerebellum (these data are not shown here). The developmental increase in enzyme activity was more or less linear in the forebrain and oB up to 20 days and in the cerebellum up to 50 days. This suggests an increase in glutamine synthetase activity which is associated with the maturation process rather than the proliferation of astrocytes. Another protein which is down-regulated is glutamate dehydrogenase 1 (Glud1) which catalyzes the reversible interconversion between glutamate and α-ketoglutarate. Glutamate serves as an important metabolite in the central nervous system [94] and is present in high concentrations in the brain [95-97]. The development of glutamate dehydrogenase activity in the whole brain has been shown to be low immediately post partum, however, its activity increases towards the adult brain [98-100]. Furthermore, as Leong et al. [101] described, the general pattern of glutamate dehydrogenase activity development correlates inversely with the decreasing ammonia concentrations observed in the rat brain as the animal gets older, suggesting a role for this enzyme in ammonia detoxication. Glutamate dehydrogenase may also be potentially required in the maintenance of glutamate and, by further metabolism of γ-amino butyric acid, in neurotransmitter function, whereas this property varies not only with the age of the animal but also with the region of the brain.

Differential protein expression was also observed in aged rat brains (P637). The main differences in the expression concerned the categories of proteins of the energy metabolism, proteins of the biosynthesis, regulatory proteins, chaperones and structural proteins.

As described in the results, the expression of 3 proteins involved in the energy metabolism displays an up-regulation (e.g. creatine kinase B chain (Ckb) and cytochrome c oxidase subunit 5B (Cox5b)). The protein ATP synthase subunit alpha (Atp5a1) is down-regulated towards P90. As stated by Villa et al. [102], there is clear evidence that the oxidative energy metabolism gradually decreases with aging and the capacity of the brain to meet stress conditions is reduced with advancing age. Also Turpeenoja et al. [103] described that mitochondria from old animals contain less respiratory units than those from young animals and that the energy transduction capability is impaired by aging. They could observe two inner membrane proteins (cytochrome b and subunit II of cytochrome oxidase) which showed an age-related increase and they assumed the abundance of these proteins may be related to a decreased degradation rate leading to the accumulation of some polypeptides, including abnormal and inactive forms of proteins. This might cause partial inactivation of some enzymes with a concomitant decline in their specific activity. It has been postulated that senescent cells have reduced capacity to selectively identify and degrade defective proteins [104] with the consequence of accumulating functionally inactive polypeptides.

The proteins involved in protein biosynthesis reveal a higher expression (members of the heterogeneous nuclear ribo-nucleoprotein family (heterogeneous nuclear ribonucleoprotein F (Hnrnpf), heterogeneous nuclear ribonucleoprotein K (Hnrnpk)) at P637 in comparison to P90. As stated above, these proteins are involved in mRNA metabolism, DNA-related functions and microRNA biogenesis. This also includes processing in alternative splicing during neuronal differentiation [52]. As stated by Tollervey et al. [105], especially genes with metabolic functions show expression changes during aging which is linked with alternative splicing. This indicates that alternative splicing complements the transcriptional regulation in modifying the molecular machinery for the repair of oxidative DNA damage in the brain [106,107].

Also regulatory proteins exhibit major changes in the expression at P637 versus P90; the protein Rab GDP dissociation inhibitor alpha (Gdi1), for example, is up-regulated at this stage as well as at P7 compared to P90. This protein is a regulator of transport processes such as vesicular trafficking, docking off transport vesicles with their corresponding acceptor membranes [108] which is important at early stages of development. The higher expression of this protein at later stages, in this case at P637, could be inferred as an effect leading to endoplasmic reticulum-associated protein degradation by either the proteasome or macroautophagy pathway at higher age [109]. However, this expression pattern could not be shown for Rho GDP-dissociation inhibitor 1 (Arhgdia) and annexin A5 (Anxa5)) which show a down-regulation at P637. The calcium-binding protein 39 (Cab39) could not be detected at this developmental stage. As Iacopino et al. [110] described with the analysis of decreased expression of 28 kDa calbindin-D, another calcium-binding protein, in 27-month-old rat brain tissue, aging in both the rat and human central nervous system is accompanied by increased intracellular levels of free calcium, reduced activity of Ca^2+^/Mg^2+^-ATPase, and a decreased ability of the mitochondria to sequester calcium [111,112]. This altered calcium homeostasis is affected by both normal aging and various neurodegenerative diseases.

Moreover, the group of chaperones contains proteins which have a differential expression at the analyzed developmental stages. For example, two members of the heat shock protein 90 family (HSP 90-alpha (Hsp90aa1), heat shock protein HSP 90-beta (Hsp90ab1)) show an up-regulation towards P90. As described by Proctor et al. [113], neurodegeneration is an age-related disorder which is characterized by the accumulation of aggregated protein, loss of protein homeostasis and neuronal cell death. Two central systems in protein homeostasis are the chaperone system (includes Hsp90), which promotes correct protein folding, and the cellular proteolytic system, which degrades misfolded or damaged proteins. A stochastic model of Hsp90 [114] showed that under conditions of low or transient stress, chaperone capacity is sufficient to maintain protein homeostasis. Even under conditions of increasing stress with age, normal chaperone capacity is able to process the increasing overload due to the mechanism of up-regulation of HSPs after stress. This observation could also be the reason for the up-regulation of the proteins in our analysis. However, other proteins (T-complex protein 1 subunit beta (Cct2)) which are also involved in chaperone activity show a down-regulation towards P90 or are absent at this stage (protein disulfide-isomerase A3 (Pdia3)). Also Vanguilder and Freeman [115] mentioned a decreased expression of these proteins as an example for age-related dysregulation, which indicates a loss of protein quality regulation that may contribute to a buildup of cytoskeletal proteins (e.g., increased neurofilament light chain and tubulin) with increasing age.

The category of structural proteins showed 7 up-regulated proteins (actin beta (Actb), myristoylated alanine-rich C-kinase substrate (Marcks) as well as several members of the tubulin family (tubulin alpha-1A chain (Tuba1a), tubulin beta-3 chain (Tubb3), tubulin beta 4A (Tubb4)). It could be hypothesized that the upregulation of these structural proteins might be due to the cytoskeletal dynamics in age-related neuronal dysfunction which was mentioned above. An indicator for this circumstance could also be the down-regulation of other structural proteins (lamin-B1 (Lmnb1) and septin-11 (Sept11)) towards P90. This means, by up-regulating the expression of established structural proteins, the organism tries to counteract the loss of essential neuronal key elements during aging by maintaining the functional organization of the cytoskeleton in general, for example.

**Figure 7:**
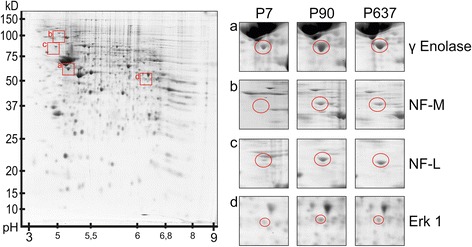
**Left side: overview of a control gel (P90) with rectangles (a-d) around regions of spots with differentially regulated proteins.** Right side: Magnification of the marked regions **(a-d)** for each developmental stage (P7, P90, P637). Each spot is marked by a circle.

Five differentially expressed proteins were validated in Western Blots (Figure 6). In comparison to the 2DE-analysis (Figure 7), the same developmental expression changes of these proteins were detectable. All of these proteins are involved in generating and regulating cell processes, axons, dendrites and also processes of glial cells.

Beyond that it could be established that additional differentially expressed proteins of the three developmental stages are involved in proliferation, migration and differentiation. Protein-members of the energy and amino acid metabolism, for example, as well as the structural proteins show that the maintenance of the oB with substrates differs during the development of the rat brain. Some of these proteins could be candidates for further investigation regarding the survival and differentiation of transplanted progenitor cells in neonatal and adult striata. This also gives the option to connect the differentially expressed proteins to their specific metabolic pathways in future studies and to analyze factors and candidates which influence the differentiation of neuronal progenitor cells.

Furthermore, a differential expression of proteins was also detectable in brains of adult and aged rats (P90, P637). This could be an indicator for the capability of the oB to still generate and integrate cells of the nervous system by the RMS of the SVZ in adulthood. In general, the oB is of special interest as it reveals spontaneous neurogenesis throughout the entire lifetime, suggesting that it plays a functional role in physiological cell replacement in aging, learning and cognition, as well as proposing a therapeutic potential in neurological diseases [13,116], including neurodegenerative disorders like Alzheimer’s and Parkinson’s disease.

## Conclusions

In conclusion, the proteomic analysis of the oB at three different developmental stages (P7, P90, P637) indicates several changes of protein expression including up- and down-regulation of single proteins or the appearance of proteins at specific developmental stages only, supporting the concept of an extraordinarily high plasticity of this part of the rat brain.

Yet, the performed differential 2D gel spot abundance analysis does not exclude the simultaneous presence of proteins/isoforms with no detectable abundance differences.

However, the analysis may contain the same proteins other than that identified as differential with regards to its abundance. As listed in the results, some abundant proteins also show an absence at single developmental stages which may result from different posttranslational modifications. However, the aim of the present study was not to characterize structural differences of the same protein in different spots but rather to detect protein spots that differed in abundance and provide indications for the changes in protein expression during development. For the precise analysis of expression changes of single proteins, further studies are needed to verify if all individual spots of this protein were detected to determine its exact differential regulation. This also includes spots which are located in regions of the gel that show a lower resolution, for example.

In this study, a relative small amount of proteins is considered to be expressed differentially by applying a restricted SVQ of greater 1.67 and smaller 0.6. An additional analysis of extensive SVQ ranges (e.g. P0, P20, P40, P200) could give a more detailed view about the development of the oB. Furthermore, usage of additional methods (e.g. difference gel electrophoresis, DIGE) could increase the systems sensitivity, and reproducibility. Also an additional usage of purification protocols could provide a more detailed view on small proteins (e.g. neurotransmitters) and plasma membrane proteins for further analysis.

## Methods

### Treatment

Male Wistar rats (Rattus norvegicus, Charles River, Sulzfeld, Germany) of different ages (7, 90, 637 postnatal days) with 6 animals in each group were used [117,118]. The animals were housed at 22 ± 2°C under an artificial day and night rhythm with a 12 h light–dark cycle with free access to water and standard nutrition. All animal treatment and experimental procedures were conducted in compliance with the regulations and licensing of the local authorities (Landesamt für Landwirtschaft, Lebensmittelsicherheit and Fischerei Mecklenburg Vorpommern, Germany) and the Animal Care and Use Committee of the University of Rostock. According to the European Communities Council Directive of 24^th^ November 1986 (86/609/EEC) and in accordance with the above-mentioned local authorities adequate measures were taken to minimize pain or discomfort.

### Perfusion and dissection

Perfusion was performed at defined dates (7, 90, 637 days postnatal). The animals were anesthetized with ether and killed by intraperitoneal Pentobarbital-Na^+^-injection (60 mg/kg BW). Transcardial perfusion was performed with 100 – 400 ml (bodyweight depending) cooled (4°C) 0.9% NaCl- solution. After decapitation and brain dissection, the dissected brain regions were weighed and stored at −80°C until homogenization. From pentobarbital injection to −80°C storage it took less than 5 min.

### Homogenization

Protein extraction was performed according to published standardized protocols [119,120]. The brain sections were incubated with (9 × probe mass [mg]) μL lysis buffer consisting of 7 M urea (Sigma, Steinheim, Germany), 2 M thiourea (Sigma), 4% CHAPS (Sigma), 70 mM DTT (Sigma), 0.5% Bio-Lyte Ampholytes pH 3–10 (Fluka, Buchs, Switzerland) and a mixture of protease inhibitors (Roche, Basel Switzerland) additionally enriched with (0.1× probe mass [mg]) μL Pepstatin A and PMSF (Fluka) and snap-frozen at −150°C. The samples were quickly thawed and transferred into a 2 mL Wheaton potter (neo-lab, Heidelberg, Germany) for homogenization. Glass beads (Roth, Karlsruhe, Germany) were added to the suspension, following a 15 s sonication, 15 s vortexing, which was repeated six times and finished by shock freezing the suspension at 150°C. The samples were quickly thawed. They were put in a beaker on a magnetic stirrer that was filled with ice water for 15 min. Finally, the samples were centrifuged at 17.860 × g for 20 min at 4°C. The supernatant was very carefully removed using a 2 ml syringe (Becton Dickinson, Heidelberg, Germany) with a 0.5 × 25 mm needle (Becton Dickinson), because of a thick lipid coverage derived from myelinated nerve fibers. The protein concentration of the supernatant was determined by the Bradford assay.

### Two dimensional polyacrylamide gel electrophoresis (2DE)

#### Rehydration

The first dimension was performed in a PROTEAN IEF cell system (Bio-Rad, CA, USA). Protein extracts of 1 mg protein were loaded on immobilized pH 3–10 nonlinear gradient strips with a length of 17 cm (GE-Healthcare, Art.: 17-1235-01) and actively rehydrated with 300 μL rehydration buffer consisting of 6 M urea (Sigma), 2 M thiourea (Sigma), 2% CHAPS (Sigma), 16 mM DTT (Sigma), 0.5% Bio-Lyte Ampholytes pH 3 10 (Fluka) at 50 V for 12 h at 20°C.

#### First dimension: isoelectric focussing

After rehydration, electrode wicks (Bio-Rad) were added to reduce artifacts. Focusing started with the “conditioning step” (2 h) which subdivides in 2 sub-steps: (a) linear voltage rise to 500 V, step-hold 30 min, (b) linear voltage rise to 2500 V, step-hold 1 h. After that, the “slow voltage ramping” (2.5 h): quadratic voltage rise to 8000 V and the “final focusing”: actual process of focusing (duration: 50.000 Vhrs) were performed. During the whole IEF the temperature was constantly kept at 20°C. After focusing the strips were stored at −80°C.

#### Second dimension: polyacrylamide gel electrophoresis

Focused IPG-strips were equilibrated in two steps of 30 min each in 5 mL of freshly prepared SDS equilibration solution consisting of 1.5 M Tris–HCl pH 8.8 (Roth), 6 M urea (Sigma), 30% Glycerol (Sigma), 2% SDS (Sigma), trace of bromophenol blue (Roth) supplemented with 10 mg/mL DTT and 40 mg/mL iodoacetamide.

The strips were transferred on 12% homogeneous self-cast sodium dodecyl sulfate polyacrylamide gels (200 mm × 250 mm × 1.5 mm). They were run at 125 V per gel (Power Pac 1000, Bio-Rad) in the PROTEAN Plus Dodeca Cell (Bio-Rad). A cooling device (Julabo F10, Julabo Labortechnik, Seelbach, Germany) was used to ensure a constant buffer-temperature of 10°C.

#### Fixation and staining

Fixation was performed with acetic acid-methanol-solution (45% methanol, 1% acetic acid overnight. Staining of the gels was performed in a colloidal CBB G250 solution (1 g/1000 ml) (Roth) as described previously [121]. After 24 h, the gels were destained with ultra pure water and were held in cold storage (4°C) with ultra pure water until digitization.

### Gel analysis

#### Digitization

The stained gels (n = 6) were scanned as 12 bit gray scale tif-images with a F4100 scanner (Heidelberg, Heidelberg, Germany) at 300 dpi resolution. Gels were rinsed in 0.02% sodium azide (Aldrich Chemie, Steinheim, Germany), shrink-wrapped in plastic and stored at 4°C until picking for MALDI-TOF-MS.

#### Digital gel processing

For 2DE-gel image analysis, the software package Progenesis PG200 Version 2006 (Nonlinear Dynamics Ltd., Newcastle upon Tyne, U.K.) was used. Each developmental stage (P7, P90, P637) consisted of six gel images. For P90 and P637, each gel image presents a single dissected brain region, whereas in P7 the samples were pooled for 2DE. The gels were registered to a reference gel (the gel which contained most spots with highest separation and staining quality and least artifacts) and manually edited spots were matched to allow comparability of all gels.

#### Determination of differentially abundant protein spots

Protein spots in 2DE were quantified by normalizing spot volumes using the Progenesis PG200 and spot volume differences were calculated. After the comparison of the normalized gray value-spot volumes of all generated spot pairs with Access and Excel (Windows, Microsoft), the comparison of the spot volumes was determined by calculation of the spot volume quotient (SVQ, P90/P7 or P90/P637). Only those spots were considered to be significantly up- or down-regulated that showed a SVQ (Spot Volume Quotient) of 0.6 or less and 1.67 or greater. The differences were evaluated significantly if differentially expressed spots in at least four gel images (correlated spots) were detected which belong to one test group. If multiple spots of one protein were detected by mass spectrometric analysis, the differential expression was determined by the mean of their individual expression levels. If the differential expression tendency was ambiguous, the protein expression is marked as “up-down” (see Additional file 1: Table S1). The classification of the differentially expressed proteins in their respective functional protein groups itself was generated by a comparison of the proteins properties; in addition, the basic function of each protein is briefly described (according to Entrez Gene, GeneCards, UniProtKB/Swiss-Prot, and/or UniProtKB/ TrEMBL).

#### Mass spectrometric analysis of protein spots

Protein spots were excised from the gels with a spot picker (Flexys Proteomics picker, Genomic Solutions, Ann Arbor, MI, USA), transferred into 96-well plates, and subjected to in-gel digestion with trypsin. The gel plugs were washed twice with 30% acetonitrile (ACN) in 25 mM ammonium bicarbonate and 50% ACN in 10 mM ammonium bicarbonate, respectively, shrunk with ACN, and dried at 37°C. The dried gel plugs were re-swollen with 5 μl protease solution (sequencing grade trypsin, 10 ng/μl in 3 mM Tris–HCl, pH 8.5, Promega, Madison, WI, USA) and incubated for 8 h at 37°C. Thereafter, 5 μl of extraction solution (0.3% trifluoroacetic acid, 50% ACN) were added and the samples were agitated at room temperature for 30–60 min before the peptide extracts were transferred into the 96-well collection plates. The resulting peptide-containing solution was prepared for MALDI analysis by spotting 0.6 μl of the tryptic digest and 0.45 μl of matrix solution consisting of 9 mg/ml α-cyano-4-hydroxy-cinnamic acid (CHCA) in 50% ACN, 0.1% trifluoroacetic acid on standard stainless steel MALDI plates. MALDI-MS analysis was performed on a 4700 Proteomics Analyzer MALDI-TOF/TOF mass spectrometer (Applied Biosystems, Foster City, CA, USA). All acquired spectra were processed using 4700 Explore™ software (Applied Biosystems). For protein identification, spectra were submitted to MASCOT (version 2.4.0, Matrix Science, London, UK) via the MASCOT Deamon. Searches were performed against the subset of rat proteins of the UniProtKB protein sequence database (2012_01; 42755 sequences from Rattus). A mass tolerance of 60 ppm and 1 missing cleavage site were set, oxidation of methionine residues was considered as variable modification, and carbamidomethylation of cysteines as fixed modification. Peptide masses of trypsin autoproteolysis products and matrix-derived peaks were excluded. Identifications with Mascot scores greater than 59 were considered significant (p < 0.05). All results were examined carefully for reliability and occurrence of multiple proteins in the same sample.

#### Immunoblot analysis

The homogenated protein samples of the oB were dissolved in sample buffer containing 1% SDS and boiled for 10 min. Protein contents in the samples were determined according to Neuhoff et al. [122]. For immunoblotting, total cellular extracts (10–50 μg per lane) were separated by SDS-Page using 7,5% polyacryamide gels and transferred to PVDF membranes (0.2 mm, Bio-Rad, Munich, Germany). The blots were blocked with 5% non-fat dry milk powder in TBS for 30 min and incubated with the individual primary antibodies. The following antibodies were used (dilutions are given in the brackets): rabbit polyclonal anti-Gamma-enolase (1:500), mouse monoclonal anti –α Tubulin (1:1000), goat monoclonal anti-neurofilament medium (1:2000), mouse monoclonal anti-neurofilament low (1:500), rabbit polyclonal anti-Erk 1,2 (1:500) and mouse monoclonal anti-Erk 1,2 P(1:500). After washing, membranes were incubated with secondary HRP-conjugated anti-mouse (1:2000), anti-goat (1:2000) and anti-rabbit (1:2000) IgG, and visualized by the enhanced chemiluminescence (ECL) procedure as described by the manufacturer (Thermo scientific, Pierce, Rockford, USA).

## Additional file

Additional file 1:
**Some differentially expressed proteins of the categories of P7 P90 and P637 which are not listed in the results are presented and described.** In **Table S1.** Remaining categories of differentially expressed proteins of P7 in comparison to P90. **Table S2.** Remaining categories of differentially expressed proteins of P637 in comparison to P90.

## Abbreviations

A: Axon
CBB: Coomassie blue
Cpg: Cytoplasmic granule
Cts: Centrosome
Cr: Chromosome
Csk: Cytoskeleton
cp: Chaperones
Cpm: CYtoplasm
Cpv: Cytoplasmic vesicle
Cts: Cytosol
ER: Endoplasmic reticulum
Eds: Endosome
Exr: Extracellular region
Gc: Growth cone
Gj: Gap junction
Golgi: Golgi apparatus
hGc: Heterotrimeric G-protein complex
La: Lipid-anchor
Lyso: Lysosome
M: Membrane
Micro: Microsome
Mmt: Mitochondrial matrix
Mm: Mitochondrial membrane
Mim: Mitochondrion inner membrane
MimS: Mitochondrion intermembrane space
Mom: Mitochondrion outer membrane
Mel: Melanosome
Mito: Mitochondrion
MT: Microtubule
Nc: Nucleus
Nf: Neurofilament
Nm: Nucleus matrix
Np: Nucleoplasm
P: Proteasome
pa: Proteins antioxidants
paam: Proteins amino acid metabolism
pb: Proteins biosynthesis
pcm: Proteins carbohydrate metabolism
pd: Proteins degradation
Per: Peroxisome
pem: Proteins energy metabolism
Pema: Proteinaceous extracellular matrix
pfm: Proteins fat metabolism
PMp: Peripheral Membrane protein
pr: Proteins regulation
pst: Proteins signal transduction
ptm: Proteins transmitter metabolism
Rs: Ribosome
S: Synapse
Sc: Secreted
Scc: Spliceosomal complex
sER: Smooth endoplasmic reticulum
sp: Structural proteins
Sv: Synaptic vesicle
Ss: Synaptosome
SR: Sarcoplasmic reticulum
TCA: Trichloroacetic acid
tp: Transport proteins
Ulc: Ubiquitin ligase complex
