# Supplementary material for: The proteome profiles of the olfactory bulb of juvenile, adult and aged rats - an ontogenetic study
Source: Proteome Sci. 2015 Feb 15;13:8. doi: 10.1186/s12953-014-0058-x (PMC4337183; doi:10.1186/s12953-014-0058-x)

In the following, the differentially expressed proteins of the categories which are not listed in the results are presented. (A: Remaining categories of differentially expressed proteins of P7 in comparison to P90. B: Remaining categories of differentially expressed proteins of P637 in comparison to P90.)

A

***Regulatory proteins***

The regulatory proteins encompass 8 proteins which are up-regulated and 4 down-regulated proteins towards P90. An example for the up-regulated proteins, cofilin-1 (Cfl1) presents a key molecule, directly regulating actin dynamics, severing and depolymerizing actin filaments to generate new barbed ends for an initiation of the actin polymerization. Another up-regulated protein is neuromodulin (GAP-43) which is selectively distributed to the axonal domain in developing neurons of regenerating neurites. Also the proteins rab GDP dissociation inhibitor alpha/beta (Gdi1, Gdi2) are up-regulated. These control the cycling of RAB GTPase and lead to learning and social alterations. For the down-regulated proteins, for example, members of the annexin family (annexin A3 (Anxa3), annexin A5 (Anxa5)) show a lower amount of expression compared to P90. These proteins are members of the calcium-dependent phospholipid-binding protein family and play a role in the regulation of cellular growth and in signal transduction pathways. One protein (sorcin (Sri))could be detected as absent at P7. It is a soluble calcium-binding protein that undergoes calcium-dependent translocation to cellular membranes. It may play a role in the release of calcium from storage sites in selective neurons and glial cells in the rat CPN.

***Proteins of the amino acid metabolism***

The proteins of the amino acid metabolism show 2 proteins which are up-regulated, for example the protein GMP synthase (GMPS) which is involved in the synthesis of guanine nucleotides. These are not only essential for DNA and RNA synthesis, but also provide GTP which is important for cell division. An amount of 7 proteins are down-regulated. An example for this category is glutamine synthetase (Glul), a protein which is located mainly in astrocytes of the brain. One of the primary roles of this protein is the protection of neurons against excitotoxicity by taking up excess ammonia and glutamate and converting it into glutamine. Another protein which is down-regulated is glutamate dehydrogenase 1 (Glud1) which is responsible for the reversible interconversion between glutamate and a-ketoglutarate. It is mainly expressed in glial cells, speciﬁcally in regions where a high expression of glutamatergic neurons occurs, including the cortex. An amount of 2 proteins are absent at P7 (histidine triad nucleotide-binding protein 1 (Hint1), NAD-dependent protein deacetylase sirtuin-2 (Sirt2)). The protein Sirt2 is involved in regulating neural/glial development and axonal degeneration.

***Transport proteins***

The transport proteins encompass 6 proteins which are up-regulated, 3 down-regulated proteins and 1 protein (NSFL1 cofactor p47 (NSFL1C9))which is absent at P90. For example, cytoplasmic dynein 1 intermediate chain 2 (Dync1i2) plays a role in cargo binding and is involved in cargo specificity. The protein fatty acid-binding protein (Fabp7) serves as an intracellular carrier, shuttling fatty acids from their site of synthesis or cell entry to target organelles. Both of these proteins show a greater expression at P7 in comparison to P90.

***Proteins of the energy metabolism***

For the proteins of the energy metabolism, a larger number of down-regulated proteins (7 proteins) than up-regulated proteins (1 protein) are detectable. Examples for the down-regulated proteins are the creatine kinase B-type (Ckb) and the cytochrome b-c1 complex (Uqcrfs1). The protein Ckbcatalyzes the reversible transfer of a high-energy phosphate group between adenosine diphosphate and phosphocreatine. The protein Uqcrfs1 is a component of the ubiquinol-cytochrome c reductase complex, which is a constitutent of the respiratory chain that generates an electrochemical potential coupled to ATP synthesis, needed by various energy-dependent processes such as ion pumping, neurotransmitter cycling, cell signaling, molecular synthesis and axonal transport.

***Proteins involved in signal transduction***

The proteins which are involved in signal transduction pathways display 5 proteins which are up-regulated and 3 proteins which are down-regulated towards p90. For the up-regulated proteins, members of the 14-3-3 family (14-3-3 protein epsilon (Ywhae), 14-3-3 protein theta (Ywhaq), 14-3-3 protein zeta/delta (Ywhaz)) show a greater expression at P7 compared to P90. These proteins participate in many cellular functions by binding to specific phosphorylated sites on diverse target proteins and are also important for brain development and neuronal migration. The down-regulated proteins include the mitogen-activated protein kinase 1 (Mapk1) and the olfactory marker protein (Omp) for example. Mapk1 is a member of the family of serine/threonine protein kinases. These kinases are densely expressed in neurons of mammalian brains and play a central role in cell signaling. Also by Westernblot analysis, a down-regulated expression towards P90 was detectable (figure 6, figure 7). Omp is a cytoplasmic protein and is present throughout the cytoplasm of olfactory sensory neurons and. It is abundant in the olfactory ciliary lumen. It represents a modulatory component of a detection/signal transduction cascade.

***Degratory proteins***

All of the degratory proteins are up-regulated towards P90 (7 proteins). These include members of the 26S proteasome multicatalytic proteinase complex (26S protease regulatory subunit 6B (Psmc4), proteasome 26S subunit (Psmd7), proteasome subunit alpha type-2 (Psma2), proteasome subunit alpha type-6 (Psma6), proteasome subunit beta type-7 (Psmb7)) which is involved in the ATP-dependent degradation of ubiquitinated proteins.

***Chaperones***

Also the chaperones display 4 up-regulated proteins. These include members of the heat shock protein 70 family (heat shock 70kDa protein 5 (Hspa5), heat shock cognate 71 kDa protein (Hspa8)) as well as members of the disulfide isomerase family A (protein disulfide-isomerase A3 (Pdia3), protein disulfide-isomerase A4 (Pdia4)).

***Proteins of the fat metabolism***

The proteins of the fat metabolism show a down-regulation of 3 proteins, additionally 1 protein (prostamide/prostaglandin F synthase (Fam213b)) is absent at P7. This protein is one of the most abundant prostaglandins in the brain. It catalyzes the reductions of prostamide H2 to prostamide F2α and PGH2 to PGF2α.

***Proteins with antioxidant capacity***

Proteins with antioxidant capacity exhibit a mixed expression towards P90. An amount of 2 proteins are up-regulated (N(G),N(G)-dimethylarginine dimethyl-aminohydrolase 1 (Ddah1), N(G),N(G)-dimethylargininedimethylaminohydrolase 2 (Ddah2)), 2 proteins are down-regulated (peroxiredoxin-6 (Prdx6), superoxide dismutase (Sod1)) and 1 protein (peroxiredoxin-4 (Prdx4)) is absent at P90. All these proteins are members of the major antioxidant enzymes. Especially Sod1 has major importance at the highly metabolic period of myelin sheath synthesis and during active myelin sheath formation in the developmental postnatal rat.

***Proteins of the fat metabolism***

The category of proteins which are participating in the energy metabolism reveals 3 up-regulated proteins. Ckb and cytochrome c oxidase subunit 5B (Cox5b) belong to up-regulated proteins for example. The protein Cox5b is a mitochondrial enzyme involved in the oxidative phosphorylation process in which ATP is generated for sustaining neuronal functions. Furthermore, the protein ATP synthase subunit alpha (Atp5a1) is down-regulated towards P90. Another protein (sulfite oxidase (Suox)) is absent at P90. This protein catalyzes the oxidation of sulfite to sulfate, the final reaction in the oxidative degradation of the sulfur amino acids cysteine and methionine.

B

***Chaperones***

The category of chaperones contains 2 proteins which are up-regulated (heat shock protein HSP 90-alpha (Hsp90aa1), heat shock protein HSP 90-beta (Hsp90ab1)). Both of these proteins are members of the heat shock protein 90 family which is involved in signal transduction, protein folding and degradation. The protein T-complex protein 1 subunit beta (Cct2) is down-regulated towards P90. It is a selective molecular chaperone in tubulin biogenesis, by that nascent tubulin subunits are bound to t-complex polypeptide 1 and released in assembly competent forms. One protein is absent at P637 (protein disulfide-isomerase A3 (Pdia3)). This protein interacts with lectin chaperones calreticulin and calnexin to modulate the folding of newly synthesized glycoproteins. One protein is absent at P90 (heat shock 70 kDa protein 1A/1B (Hspa1a)).

***Proteins involved in signal transduction***

The proteins which are involved in the pathways of the signal transduction show 1 protein which is up-regulated (mitogen-activated protein kinase 1 (Mapk1)). This protein which functional properties are described earlier shows a down-regulated expression at P7 in comparison to P90 (also detectable by Westernblot analysis, figure 6, figure 7). A higher expression at P637 in comparison to P90 could also be shown by Westernblot analysis, at least for thephosphorylated form of the protein (figure 6). Furthermore, 2 down-regulated proteins (14-3-3 protein beta/alpha (Ywhab), 14-3-3 protein zeta/delta(Ywhaz)) could be analyzed. Both proteins belong to the 14-3-3 family. One protein is absent at P637 in comparison to P90 (guanine nucleotide-binding protein G(o) subunit alpha (Gnao1)). This protein can interact with GAP43 which plays a role in neurite extension.

***Proteins of the carbohydrate metabolism***

The proteins of the carbohydrate metabolism show 1 protein which is upregulated (pyruvate dehydrogenase E1 component subunit alpha (Pdha1)). This protein catalyzes the overall conversion of pyruvate to acetyl-CoA and CO_2._ An amount of 2 proteins displays a down-regulation in this category (2-oxoglutarate dehydrogenase (Ogdh), 3-mercaptopyruvate sulfurtransferase (Mpst)).

***Transport proteins***

Also the transport proteins present respectively 1 up-regulated protein and 1 protein which is down-regulated in this category. The up-regulated protein (alpha-tocopherol-associated protein (Sec14l2)) belongs to the family of lipid-binding proteins. Furthermore, the down-regulated protein (calretinin (Calb2)) is a mature interneuron marker.

***Degratory proteins***

The proteins of degradation reveal 1 protein which is up-regulated and 1 protein which is absent at P90 (26S protease regulatory subunit 7 (Psmc2)). This protein is a member of the 26S proteasome multicatalytic proteinase complex.

***Proteins with antioxidant capacity***

The antioxidants possess only 1 protein which is up-regulated and 1 protein which is down-regulated towards P90. The up-regulated protein (superoxide dismutase (Sod1)) has a lower expression at P7 towards P90 where as at P637, its expression is higher than at P90.

***Proteins of the transmitter metabolism***

The category of transmitter proteins shows only a down-regulation of 1 protein (Succinate-semialdehyde dehydrogenase (Aldh5a1)).

***Proteins of the amino acid metabolism***

The last category, the proteins of the amino acid metabolism present 1 protein (glutamine synthetase (Glul)) which is up-regulated in comparison to P90. This protein, which function is described above, shows at P7 a down-regulation in comparison to P90. At P637, its expression is higher than at P90.

Suppl. Tab. 1: Proteins identified by peptide mass fingerprinting in spots from Coomassie-stained gels (see Supplementary Fig. 1) arranged by their function. Analyzed proteins which show a differential expression in both developmental stages are listed one below the other (Spot Data P7/Spot Data P637). Acc.: Accession: Accession number from UniProtKB/Swiss-Prot; Name ID: Entry name from UniProtKB/Swiss-Prot; Gene symbol: from UniProtKB/Swiss-Prot; Fold P7/FoldP637: differential expression in comparison to P90; H: number of differential expressed spots per fraction; Control / PD: spot volume quotient; Score: Mascot MOWSE-score; MW: Theoretical molecular mass (Da); pI: theoretical pI value taken from the Mascot report; Qm: Number of mass values (tryptic peptides) assigned to the identified protein; *: Spots marked with [+] contain multiple proteins which are additionally presented in Suppl. Tab. 2. The full description for the cellular location is listed in the List of abbreviations.

Suppl. Tab. 2: Proteins identified by peptide mass fingerprinting in spots from Coomassie-stained gels arranged by their function. This table shows only those spots which are marked by [+] in Suppl. Tab. 1. Each protein belongs to a group of proteins of at least one spot (multiple proteins in a single spot). The full description for the abbreviations is described in the legend of Suppl. Tab. 1.

Suppl. Fig. 1: Annotation of accession numbers from Swiss Prot in the reference gel (P90, reference gel: Bulbus 5-4). Protein mixes and multiple protein presentations in different spots can be derived from branching lines. A: differential expression of P7 in comparison to P90 (green: down-regulated proteins, black: up-regulated proteins); B: differential expression of P637 in comparison to P90 (green: down-regulated proteins, pink: up-regulated proteins).

A


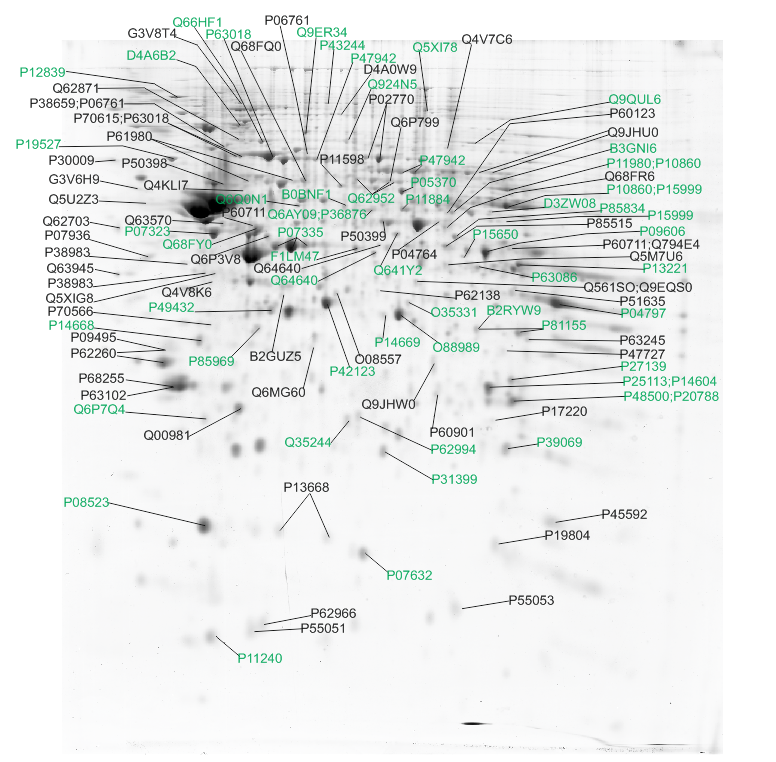


B


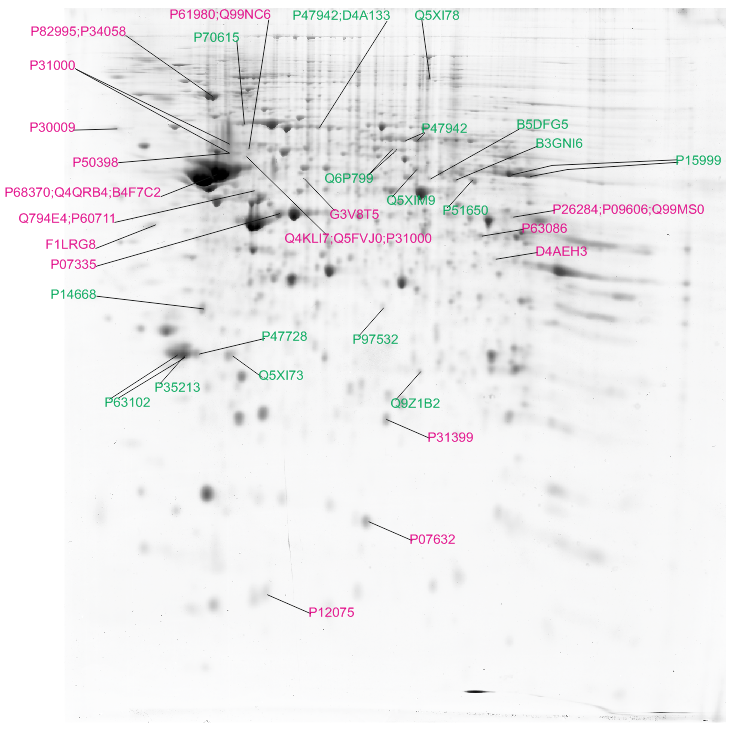

Supplement: Additional file 1: — Some differentially expressed proteins of the categories of P7 P90 and P637 which are not listed in the results are presented and described. In Table S1. Remaining categories of differentially expressed proteins of P7 in comparison to P90. Table S2. Remaining categories of differentially expressed proteins of P637 in comparison to P90. [file 12953_2014_58_MOESM1_ESM.docx]
